# Supplementary figures and images for: Identification of Key Genes Related to Dormancy Control in Prunus Species by Meta-Analysis of RNAseq Data
Source: Plants (Basel). 2022 Sep 21;11(19):2469. doi: 10.3390/plants11192469 (PMC9573011; doi:10.3390/plants11192469)

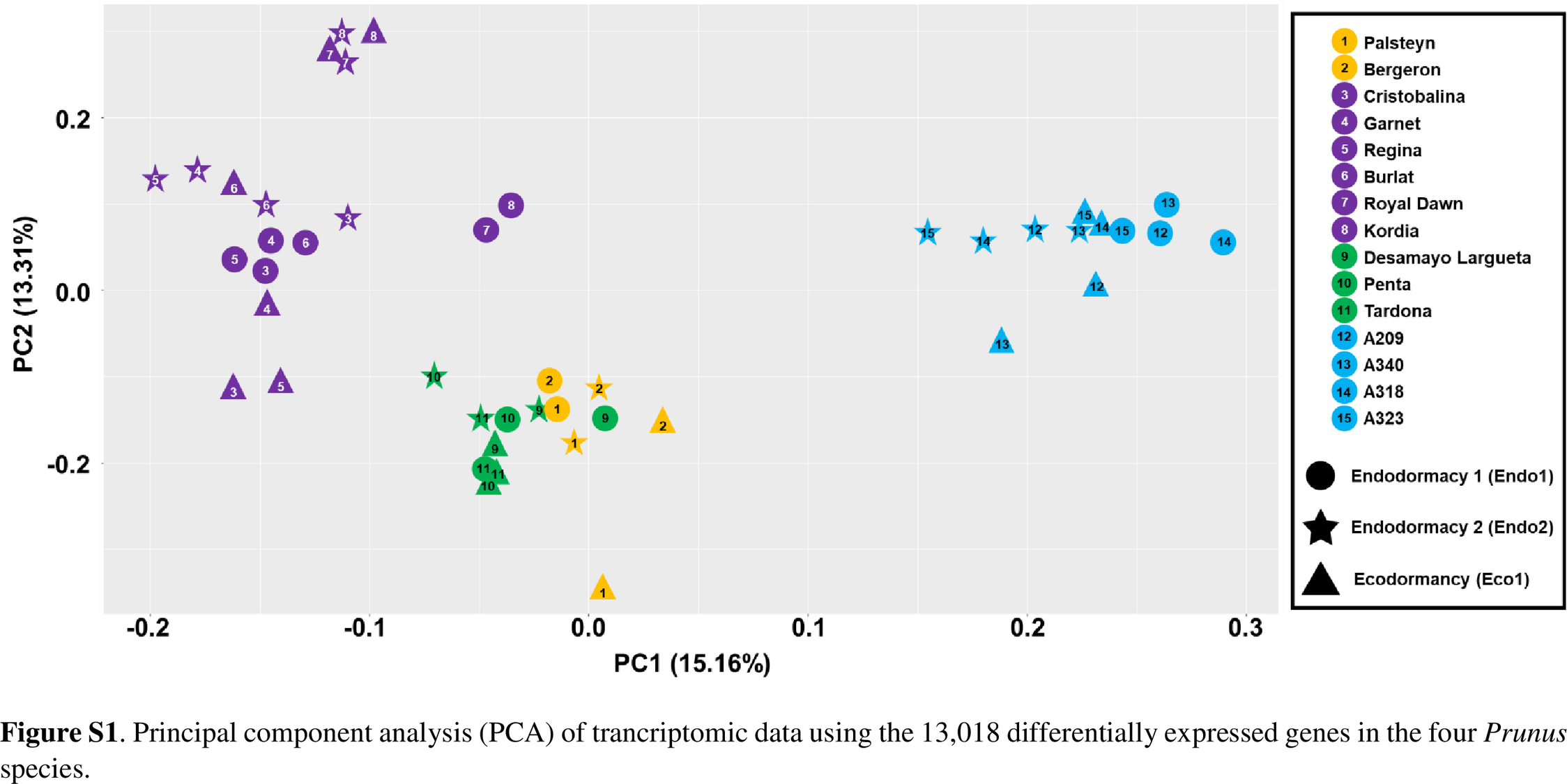

Supplement: Supplementary file 1 [file plants-11-02469-s001.zip › Figure S1.tif]

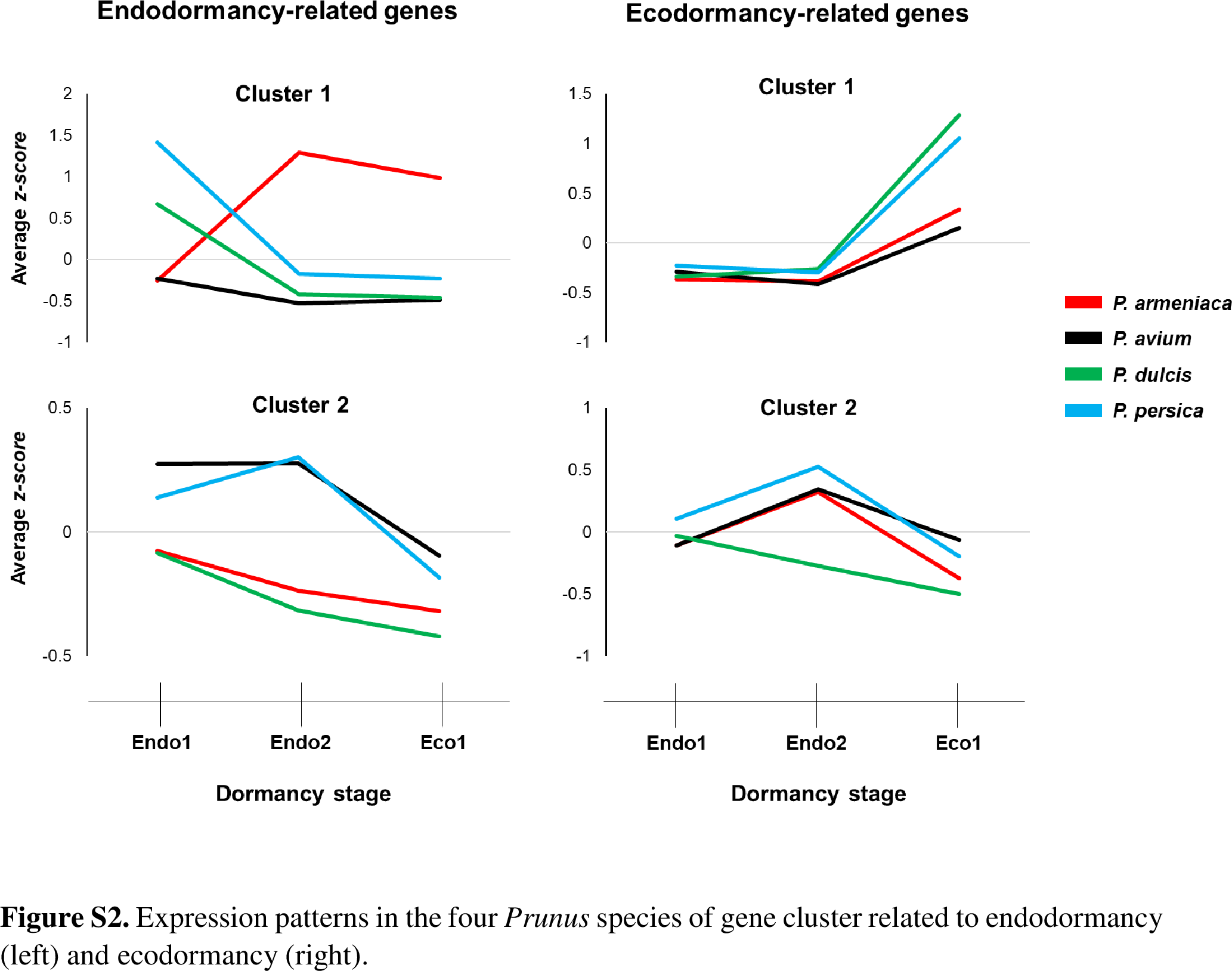

Supplement: Supplementary file 1 [file plants-11-02469-s001.zip › Figure S2.tif]
